# Mortality by cryptococcosis in Brazil from 2000 to 2012: A descriptive epidemiological study

**DOI:** 10.1371/journal.pntd.0007569

**Published:** 2019-07-29

**Authors:** Emmanuel Alves Soares, Márcia dos Santos Lazera, Bodo Wanke, Marcela de Faria Ferreira, Raquel Vasconcellos Carvalhaes de Oliveira, Adeno Gonçalves Oliveira, Ziadir Francisco Coutinho

**Affiliations:** 1 Natan Portela Institute of Tropical Diseases, State Secretary of Health of Piauí, Departament of infectious disease, Teresina-PI, Brasil; 2 National Institute of Infectious Diseases Evandro Chagas, Oswaldo Cruz Foundation, Department of Mycology, Rio de Janeiro-RJ, Brazil; 3 Health Center Germano Sinval Farias, National School of Public Health/Oswaldo Cruz Foundation, Department of Public Health, Rio de Janeiro-RJ, Brazil; National Institute for Communicable Diseases, Johannesburg, South Africa, SOUTH AFRICA

## Abstract

**Background:**

Cryptococcosis is a neglected and predominantly opportunistic mycosis that, in Brazil, poses an important public health problem, due to its late diagnosis and high lethality.

**Methods:**

The present study analysed cryptococcosis mortality in Brazil from January 2000 to December 2012, based on secondary data (Mortality Information System/SIM-DATASUS and IBGE).

**Results:**

Out of 5,755 recorded deaths in which cryptococcosis was mentioned as one of the morbid states that contributed to death, two distinct groups emerged: 1,121 (19.5%) registered cryptococcosis as the basic cause of death, and 4,634 (80.5%) registered cryptococcosis associated with risk factors, mainly AIDS (75%), followed by other host risks (5.5%). The mortality rate by cryptococcosis as the basic cause was 6.19/million inhabitants, whereas the mortality rate by cryptococcosis as an associated cause was 25.19/million inhabitants. Meningitis was the predominant clinical form (80%), males were the more affected (69%), and 39.5 years old was the mean age. The highest mortality rate due to cryptococcosis as basic cause occurred in the state of Mato Grosso (10.96/million inhabitants). Mortality rates due to cryptococcosis as associated cause were highest in the states of Santa Catarina (70.41/million inhabitants) and Rio Grande do Sul (64.40/million inhabitants), both in the South Region. Southeast, Northeast and South showed significant time trends in mortality rates.

**Conclusions:**

This study is relevant because it shows the magnitude of cryptococcosis mortality linked to AIDS and removes the invisibility of a particular non-AIDS-related disease, accounting for almost 20% of all cryptococcosis deaths. It can also contribute to control and surveillance programs, beyond highlighting the urgent prioritization of early diagnosis and proper treatment to reduce the unacceptable mortality rate of this neglected mycosis in Brazil.

## Author summary

Cryptococcosis is an invasive, global, and neglected mycosis. Species of the *Cryptococcus neoformans* complex cause opportunistic infections in immunosuppressed hosts, particularly AIDS patients, while infections by species of the *C*. *gattii* complex predominate as a primary endemic mycosis in tropical and subtropical areas. In Brazil, it is an important and hidden public health problem, mainly in its meningitis form, whose lethality ranges from 45 to 65%, but remains as a not-notifiable disease. Brazilian studies placed it as the first cause of mortality among all AIDS-associated systemic mycoses and the second cause of mortality among systemic mycoses in general. This national study used data from the Brazilian Mortality Information System (SIM) in which all mentioned causes were considered, allowing the analysis of the associated causes of deaths and showing two different patterns of infections: a primary and an opportunistic cryptococcosis. Primary cryptococcosis presented a peculiar epidemiological regional profile and the opportunistic cryptococcosis was hidden by several immunosuppressive conditions. The authors expect that this study can support a better understanding of this infection and encourage more research and public health initiatives to prevent and control the cryptococcosis, both primary and opportunistic.

## Introduction

Encapsulated yeasts of the *Cryptococcus neoformans/Cryptococcus gattii* species complexes are the causative agents of cryptococcosis, a systemic mycosis of humans and animals, acquired by inhalation of their spores—desiccated yeast cells or basidiospores—from the environment [1,2]. Although usually regressive, some cases develop cryptococcal lung injury, which can spread to other sites or organs. On reaching the central nervous system, it may cause meningoencephalitis, the most severe form of cryptococcosis that, without early diagnosis and proper treatment, is highly lethal or disabling [3,4].

*Cryptococcus neoformans* infection predominates in immunocompromised hosts, being globally a threat to people living with HIV/AIDS, causing approximately 15% of AIDS-related annual mortality [5,6]. Cryptococcosis by *C*. *gattii* occurs mainly in otherwise immunocompetent hosts, but some immune deficiency not detected by routine tests may predispose individuals to this infection [7,8].

It is estimated that more than 300 million people worldwide, of which about 3.8 million in Brazil, suffer from a serious fungal infection every year, resulting in more than 1,350,000 deaths [9,10]. Among these diseases is cryptococcosis, with an overall incidence varying from 0.04 to 12% per year among people with HIV [5].

The global incidence of cryptococcosis in people living with HIV/AIDS in 2008 was estimated in approximately 1 million meningitis cases annually (range 371,700–1,544,000) causing around 625,000 deaths [5]. The highest number of yearly cases was estimated to occur in sub-Saharan Africa (720,000), followed by South–East Asia, and Latin America as the second and third regions with the most cases of cryptococcal meningitis (54,400) [5].

Since then, due to extensive antiretroviral therapy (ARVT) expansion, AIDS-related deaths have been reduced by 45% [6]. In 2014, the global incidence cases of cryptococcal meningitis was estimated at 223,100 (95% CI 150,600–282,400) and the annual global deaths were estimated at 181,100, with 135,900 (75%; [95% CI 93,900–163,900]) deaths in sub-Saharan Africa [6]. Latin America’s annual burden of cryptococcal meningitis estimate was 5300 (2600–8900 interval) and deaths from cryptococcal meningitis were 2400 (1100–4400) [6]. But, even so, cryptococcosis is not on the WHO neglected tropical diseases list [5,11].

Besides the well-known outbreak in North America [12,13], cryptococcosis by *C*. *gattii* presents a peculiar epidemiological profile in South America, especially in Brazil, where it is endemic in large areas of the Amazon region and the semi-arid Northeast region [14–19].

However, data available on cryptococcosis in Brazil is fragmented and circumscribed, mostly based on indirect data on AIDS programs and some based on analyses of series of cases, diagnosed in regional centres. According to studies regarding mortality related to systemic mycoses in the nationally, cryptococcosis is the second cause of mortality among them [20]. Moreover, cryptococcosis is highlighted as the most frequent among the systemic mycoses associated with AIDS [21], assuming its essentially opportunistic character.

The cryptococcosis lethality rate in Brazil is substantial, reported in the range of 45% to 65% [22], independent of the presence of risk factors, dominated by association with AIDS, as well as the primary form of the disease. A different scenario is seen in developed countries, as for example in Canada, in non-HIV hosts, where the diagnosis of pulmonary forms is more frequent than meningitis, the overall lethality is about 8% and there is a control program and surveillance for primary cryptococcosis [23].

Cryptococcosis is a major public health problem in Brazil, most cases are diagnosed as central nervous clinical forms, mainly meningitis. Only a few cases are diagnosed in a pulmonary form, which usually disseminates to meningoencephalitis, increasing hospitalizations and lethality. Late diagnosis of cryptococcosis slows crucial therapeutic measures to reduce sequelae and avoid lethal outcomes. Nevertheless, cryptococcosis is not a reportable disease in Brazil, and the real magnitude of its mortality is unknown [15].

In order to improve epidemiological surveillance, regional strategies and priorities for early diagnosis and treatment of cryptococosis in HIV as well as in non-HIV groups, this study aims to characterize the mortality by cryptococcosis as a health problem with a diverse geographical pattern in Brazil. This paper shows the magnitude of cryptococcosis mortality and points to cryptococcosis as a severe and often fatal neglected mycosis in Brazil. The vast majority of deaths are hidden by several immunosuppressive conditions.

## Methods

This is a descriptive epidemiological Brazialian study, based on secondary data for the period 2000 to 2012, covering a historical series of 13 years.

The study was approved by the Ethics Research Committee of the Sérgio Arouca Brazilian National School of Public Health, number 37353614.5.0000.5240.

The research used secondary data from the DATASUS/Ministry of Health (MS) Mortality Information System (SIM) and the Brazilian Institute of Geography and Statistics (IBGE). Therefore, the individuals whose information was extracted were not identified individually. Furthermore, there was no direct intervention with the patient and / or relatives, ensuring anonymity.

The DATASUS/Ministry of Health (MS) SIM is the official source of death data for infectious and parasitic diseases (IPDs). SIM compounds the National Epidemiological Surveillance System (NESS), providing data about deaths in Brazil through information registered on death declaration (OD), including basic and associated cause, based on the 10^th^ International Classification of Disease (ICD). This data is collected by Municipal Health Secretaries (MHS) and registered in a national database and available for consultation. SIM data collection methodology did not change during the study period. Demographic data of the population and cartographic bases of the Brazilian federal units and regions were obtained from IBGE.

The following variables were considered: cryptococcosis as basic or associated cause of death, gender, age, and place of residence. Data was distributed and analyzed according to country, regions and states. Deaths were studied according to their frequency by place of residence and their temporal and spatial distribution, estimating mortality and trend coefficients and analyzing their geographical distribution.

Basic cause of death was defined as a disease or condition that initiated the chain of pathological events that led directly to death. Associated cause of death was defined as a pathological condition that had an unfavourable effect and contributed to death, mentioned in the death certificate. The classification between basic or associated cause was attributed by the physician who completed the death certificate. Only recently, data on deaths according to multiple causes is available in the mortality database.

The mean mortality rate was estimated taking as numerator the number of basic cause of death by cryptococcosis at specific locations during the study period (2000–2012). The utilized denominator was the mean size of the Brazilian population, in the same period, multiplied by 1,000,000 inhabitants. The same methodology was used for cryptococcosis as an associated cause. The mean mortality rate for all mentions was also estimated in the death certificates, that is, by the sum of both conditions above.

To highlight the particularity of cryptococcosis, the total number of times cryptococcosis was mentioned, either as the basic or associated cause, that is, the total number of mentions among the diseases that contributed to death, was used. The ratio was then estimated by dividing the frequency of cryptococcosis as a mentioned cause by frequency as the basic cause (ratio: total mentions/basic cause) [24].

In order to analyze association between gender and associated or basic cause, we used a chi-square test, with significance level of 5%. We used a Poisson model with offset term to model the mortality rate by cause (associated or basic), age groups and gender. The incidence density ratios (IDR) and 95% confidence intervals (95% CI) were obtained from this model.

The information on mortality by cryptococcosis with reference to each region or federated unit was analyzed according to its geographic distribution and presented through tables and thematic maps.

We analyzed the time trends of mortality rate by Joinpoint analysis for basic and total cause of death. For this, we modelled the rates by Poisson model with quasilikelihood estimation, in order to solve the overdispersion problem. After, we used a segmented regression to determine the breakpoints in which we observed a significant change in trend of mortality rate. The Annual Percentage Change (APC) in each trend was obtained, with a 95% confidence interval (CI). Graphs of mortality rates observed (squared points) and of mortality rates predicted by the Poisson segmented regression (lines) were provided.

Tabwin, Microsoft Excel 2010, R 3.5.1 and package segmented and QGIS were used to obtain the database, tabulation, trends and graphing.

## Results

From 2000 to 2012, a total of 5755 deaths were recorded in Brazil in which cryptococcosis was mentioned. Of these, cryptococcosis was recorded as the basic cause of death in 1121 deaths (19.5%), representing a mean mortality rate of 6.09/ million inhabitants. The remaining 4634 (80.5%) deaths from cryptococcosis were recorded as an associated cause with a mortality rate of 25.19/million inhabitants. Male deaths were more common in both the basic and associated causes (Table 1).

**Table 1:** Cryptococcosis deaths by gender and age groups, by basic and associated cause. Brazil, 2000 to 2012.

Gender/age groups	Basic cause		Associated cause						TOTAL	
			HIV+		OtherRF		Subtotal			
							associated			
Gender*	**N**	**%**	**N**	**%**	**n**	**%**	**N**	**%**	**N**	**%**
Male	698	62.3	3086	71.5	214	66.9	3300	71.2	3998	69.5
Female	423	37.7	1228	28.5	106	33.1	1334	28.8	1757	30.5
**Total**	**1121**	**100**	**4314**	**100**	**320**	**100**	**4634**	**100**	**5755**	**100**
Age groups**										
< 20	76	6.7	68	1.5	17	5.3	85	1.8	161	2.9
20 to 39	402	36.0	2605	60.4	85	26.5	2690	58.0	3092	53.7
40 to 59	418	37.3	1528	35.4	111	34.6	1639	35.4	2057	35.7
**Subtotal 20–59**	**820**	**73.3**	**4133**	**95.8**	**196**	**61.1**	**4329**	**93.4**	**5149**	**89.4**
≥ 60 years	225	20.0	112	2.7	108	33.6	220	4.8	445	7.7
**Total**	**1121**	**100**	**4314**	**100**	**320**	**100**	**4634**	**100**	**5755**	**100**

Source: DATASUS/MS

*Chi-squared Gender vs Basic or Associated cause: p-value<0.001

N: number; %: percentage; HIV+: positive serology for HIV; RF: risk factor

** Years old

The frequency rate of basic cause (mentions/basic cause) was 5.13 (5755/1121).

Of the 4314 cases associated with AIDS, 71.5% of deaths occurred in males, prevailing in the age range of 20 to 59 years old, accounting for 95.8% (n = 4133) of the deaths. In the group of other risk factors (n = 320), males represented 66.9% of the deaths (Table 1). The IDR found corroborates the increased risk of death in males, the age group of 20 to 59 years and associated cause (Table 2).

**Table 2:** Incidence Density Ratios (IDR)* for risk of cryptococcosis death in relation to basic or associated causes, age groups and gender, Brazil, 2000 to 2012.

	crude IDR (95% CI**)	adj IDR (95% CI**)
Associated vs basic cause	4.00 ((3.75,4.27)	3.91 (3.67,4.18)
Age groups (ref = 0 to 9)***		
10 to 19	4.01(2.69,5.96)	3.99 (2.68,5.94)
20 to 59	57.51(40.17,82.34)	56.14 (39.22,80.38)
≥60	28.28(19.54,40.92)	26.34 (18.2,38.11)
Male vs Female	2.28(2.15,2.41)	2.28 (2.15,2.41)

*Poisson Model with three covariates (death cause, age groups and gender) and an offset term

** Confiance Interval

***Years old

Among deaths of those younger than 20 years of age, (2.9% of the total), cryptococcosis mentioned as basic cause accounted for 6.7% (n = 76), and 5.3% (n = 17) of deaths by cryptococcosis due to other risk factors, excluding HIV+, as compared to 1.5% (n = 68) of cryptococcosis AIDS-related deaths. In the basic cause group, cryptococcosis deaths among those older than 60 represent 51% of total mentions in this age group (225/445) and among those younger than 20 years old, represent 47% of total mentions in this group (76/161) (Table 1).

Several known immunosuppressive conditions were recorded as basic cause in 80% (n = 4634) of the deaths where cryptococcosis was mentioned as associated cause. AIDS was the major immunosuppressive disorder with 75% (n = 4314 deaths), followed by other immunodeficiency conditions or risk factors with 5.5% (n = 320) of deaths: non-Hodgkin lymphoma (27), unspecified immunodeficiency (17), lymphoid leukemia (13), chronic renal failure (12) and other causes (251), reflecting the opportunistic face of this mycosis.

All clinical presentations of registered cryptococcosis have pointed to a severe disease, especially cryptococcal meningitis. Cerebral cryptococcosis–ICD (International Classification of Diaseases) B45.1 - (cryptococcal meningitis) predominated as by far the most frequent form, with 4743 deaths (82.4%) of the total mentions. In the AIDS group, this form occurred in 83.6% (3609) of deaths, whereas where cryptococcosis was the underlying cause of death, it was 79.9% (895). It is worth noting that the pulmonary form was diagnosed with cryptococcosis as a basic cause of death in 65 cases (5.8%), when associated with other risk factors 18 (5.7%) and when associated with AIDS it was recorded only in 31 deaths (0.7%) (Table 3).

**Table 3:** Deaths of cryptococcosis, according to clinical form, by basic and associated causes. Brazil, 2000 to 2012.

Clinical forms	ICD	Basic cause		Associated causes						TOTAL	
				HIV+		Other RF		Subtotal			
								Associated			
		N	%	N	%	n	%	N	%	n	%
Cerebral	B45.1	895	79.9	3609	83.6	239	74.7	3848	83.0	4743	82.4
Unspecified	B45.9	106	9.4	446	10.3	36	11.2	482	10.4	588	10.3
Disseminated	B45.7	39	3.5	174	4.0	17	5.3	191	4.1	230	4.0
Pulmonary	B45.0	65	5.8	31	0.7	18	5.7	49	1.1	114	2.0
Other forms	B45.8	16	1.4	48	1.1	7	2.3	55	1.3	71	1.2
Cutaneous	B45.2	0	0.0	6	0.1	2	0.7	8	0.1	8	0.1
Bone	B45.3	0	0.0	0	0.0	1	0.1	1	0.0	1	0.0
**Total deaths**		**1121**	**100**	**4314**	**100**	**320**	**100**	**4634**	**100**	**5755**	**100**

ICD = International Classification of Diseases; n = number; % = percentage; HIV+ = positive for HIV; Other RF = Other risk factors

The distribution of deaths and the mean mortality rate by other infectious meningitides, according to the basic cause, were also analyzed in order to assess the relevance of the central nervous system in cryptococcosis among the other meningitides.

During the study period, 21,333 meningitis deaths occurred, with a mortality rate of 115,97/million inhabitants. Among meningitis with specified cause, the meningococcal etiology was responsible for 8.6% (1,830), with a mortality rate of 9.95/million inhabitants, being the most frequent, followed by cryptococcal meningitis, with 895 deaths (4.2% of the total) and mortality rate of 4.87/million inhabitants. Also relevant were toxoplasma meningitis with 806 deaths (3.8%) and a mortality rate of 4.38/million inhabitants; viral meningitis with 753 deaths (3.5%) and a mortality rate of 4.09/million inhabitants; and tuberculous meningitis with 624 deaths (2.9%) and a mortality rate of 0.26/million inhabitants. Meningites of unknown cause were included as “other meningites” (Table 4).

**Table 4:** Deaths and mean mortality rates* for cryptococcal meningitis and other meningitis. Brazil, 2000 to 2012.

Meningitis	Deaths	%	Average rate
Meningococcal meningitis	1830	8.6	9.95
Tuberculous Meningitis	624	2.9	3.39
Other bacterial meningitis	10813	50.7	58.78
**Subtotal Bacterial meningitis**	**13267**	**62.2**	**72.12**
Cryptococcal meningitis	895	4.2	4.87
Candida meningitis	10	0.05	0.05
Coccidioidomycosis meningitis	1	0.005	0.01
**Subtotal Fungal meningitis**	**906**	**4.2**	**4.93**
Herpes virus meningitis	51	0.2	0.28
Varicella virus meningitis	9	0.04	0.05
Mumps vírus meningitis	2	0.009	0.01
Other viral meningites	691	3.2	3.76
**Subtotal Viral meningitis**	**753**	**3.5**	**4.09**
CNS infection by Toxoplasma	806	3,8	4.38
Other meningites	5601	26.2	30.45
**Subtotal Other meningites**	**6407**	**30.0**	**34.83**
**Total**	**21333**	**100**	**115.97**

Source: DATASUS/MS and IBGE

n = number; %—percentage

* = average mortality rate per 1,000,000 inhabitants

In the same period, there were 608,314 deaths from other infectious diseases listed in Chapter 1 from ICD 10. Cryptococcosis was the thirteenth cause of death between chronic and recurrent infectious disease, 1121 by basic cause. The proportion of cryptococcal deaths in the study period compared to the other infectious diseases was 0.18% (S1 Table).

**Fig 1:**
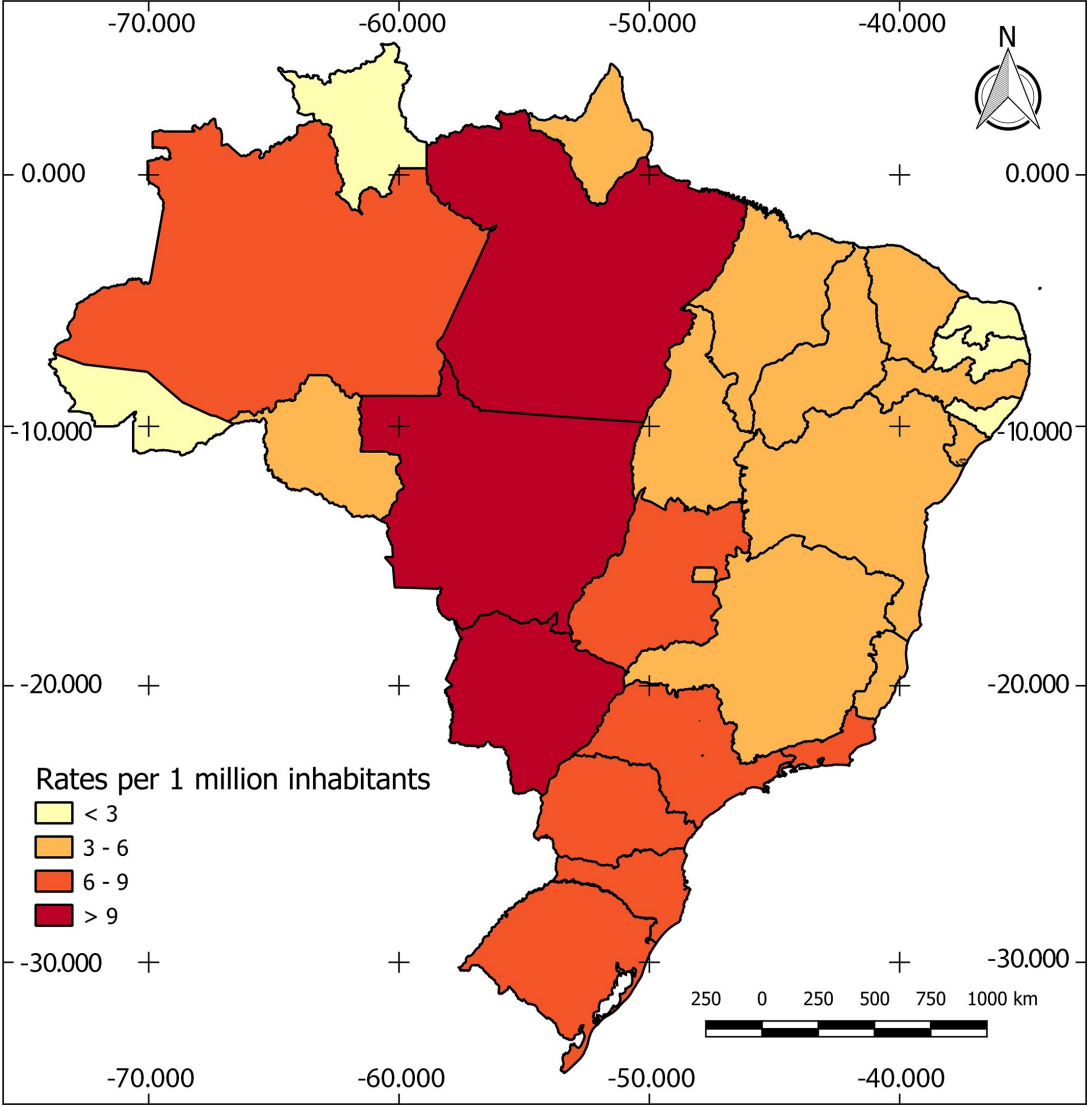
Distribution of mortality rates* by cryptococcosis (basic cause) according to States. **Brazil, 2000 to 2012.** Source: DATASUS/MS and IBGE. *: mortality rates per 1,000,000 inhabitants.

**Fig 2:**
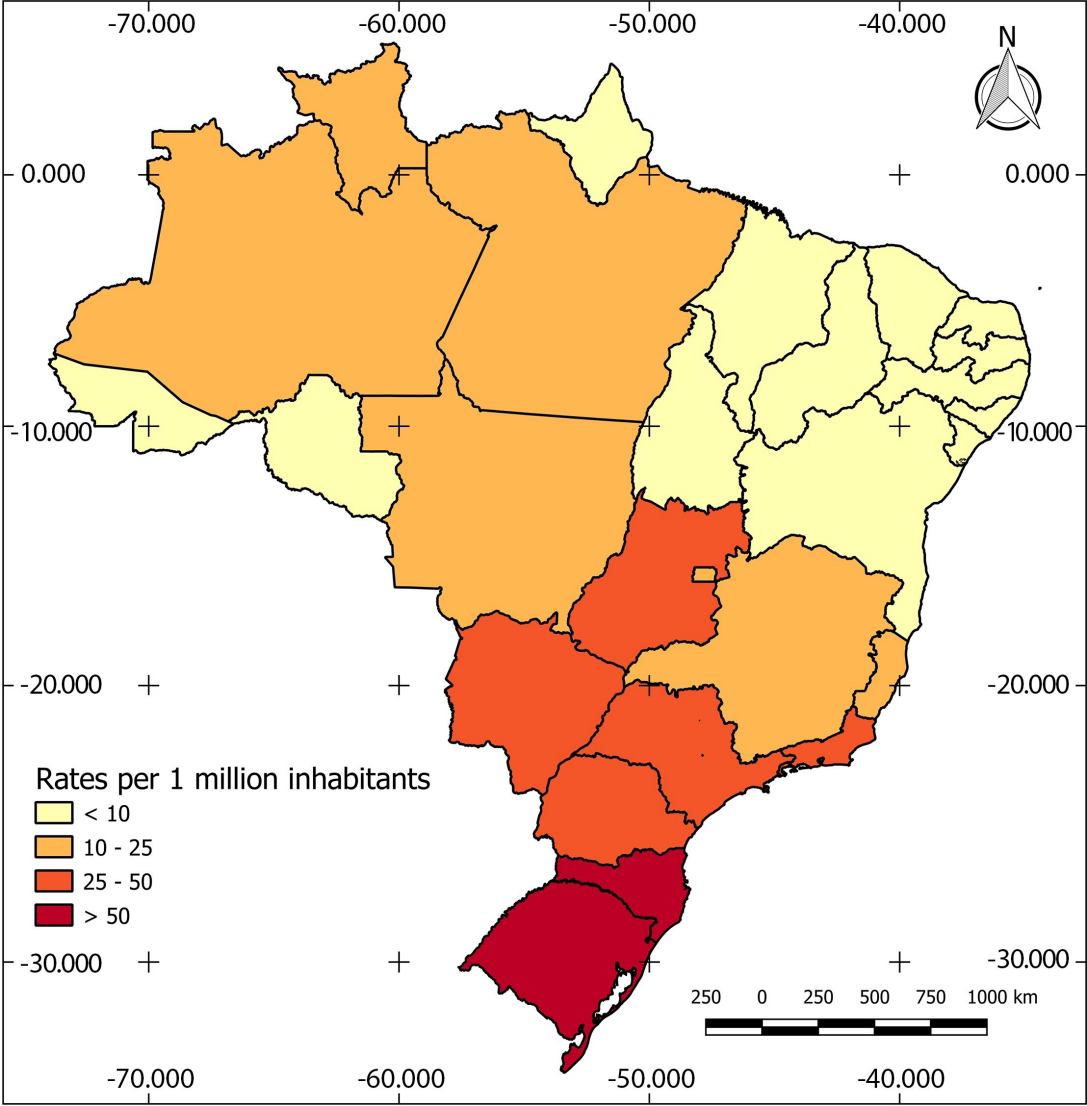
Distribution of mortality rates* by cryptococcosis (associated causes) according to States. **Brazil, 2000 to 2012.** Source: DATASUS/MS and IBGE. *: mortality rates per 1,000,000 inhabitants.

Deaths from cryptococcosis were recorded in all Brazilian states, but their distribution was not homogeneous. Thematic maps show the geographic profile of cryptococcosis mortality rates in the period, as basic cause as well as an associated cause of death (Table 5) (Figs 1 and 2).

**Table 5:** Deaths and mortality rates* by cryptococcosis, for basic and associated causes, by Regions. Brazil, 2000 to 2012.

States and Regions	Population**	Basic cause		Associated causes	
		Deaths	Mortality	Deaths	Mortality
Acre	661804	0	0.00	4	6.04
Amapá	593797	3	5.05	2	3.37
Amazonas	3237388	28	8.65	48	14.83
Pará	7033328	75	10.66	102	14.50
Rondônia	1505282	8	5.31	14	9.30
Roraima	396761	1	2.52	9	22.68
Tocantins	1292619	5	3.87	11	8.51
**NORTH**	**14720981**	**120**	**8.15**	**190**	**12.91**
Alagoas	3022824	9	2.98	17	5.62
Bahia	13836904	44	3.18	73	5.28
Ceará	8114653	30	3.70	49	6.04
Maranhão	6166395	21	3.41	21	3.41
Paraíba	3632531	4	1.10	5	1.38
Pernambuco	8465843	27	3.19	53	6.26
Piauí	3021555	18	5.96	16	5.30
Rio Grande do Norte	3017385	1	0.33	3	0.99
Sergipe	1962719	6	3.06	18	9.17
**NORTHEAST**	**51240810**	**160**	**3.12**	**255**	**4.98**
Espírito Santo	3382762	15	4.43	81	23.94
Minas Gerais	19167635	85	4.43	348	18.16
Rio de Janeiro	15422091	129	8.36	502	32.55
São Paulo	40084417	302	7.53	1419	35.40
**SOUTHEAST**	**78056905**	**531**	**6.80**	**2350**	**30.11**
Paraná	10226972	67	6.55	307	30.02
Rio Grande do Sul	10683528	90	8.42	688	64.40
Santa Catarina	5893898	51	8.65	415	70.41
**SOUTH**	**26804398**	**208**	**7.76**	**1410**	**52.60**
Distrito Federal	2373961	11	4.63	58	24.43
Goiás	5633914	38	6.74	215	38.16
Mato Grosso	2828850	31	10.96	43	15.20
Mato Grosso do Sul	2286152	22	9.62	113	49.43
**CENTRAL-WEST**	**13122878**	**102**	**7.77**	**429**	32.69
**BRAZIL**	**183945970**	**1121**	**6.09**	**4634**	**25.19**

Source: DATASUS/MS and IBGE

*: mortality rates per 1,000,000 inhabitants

** mean annual population

**Fig 3:**
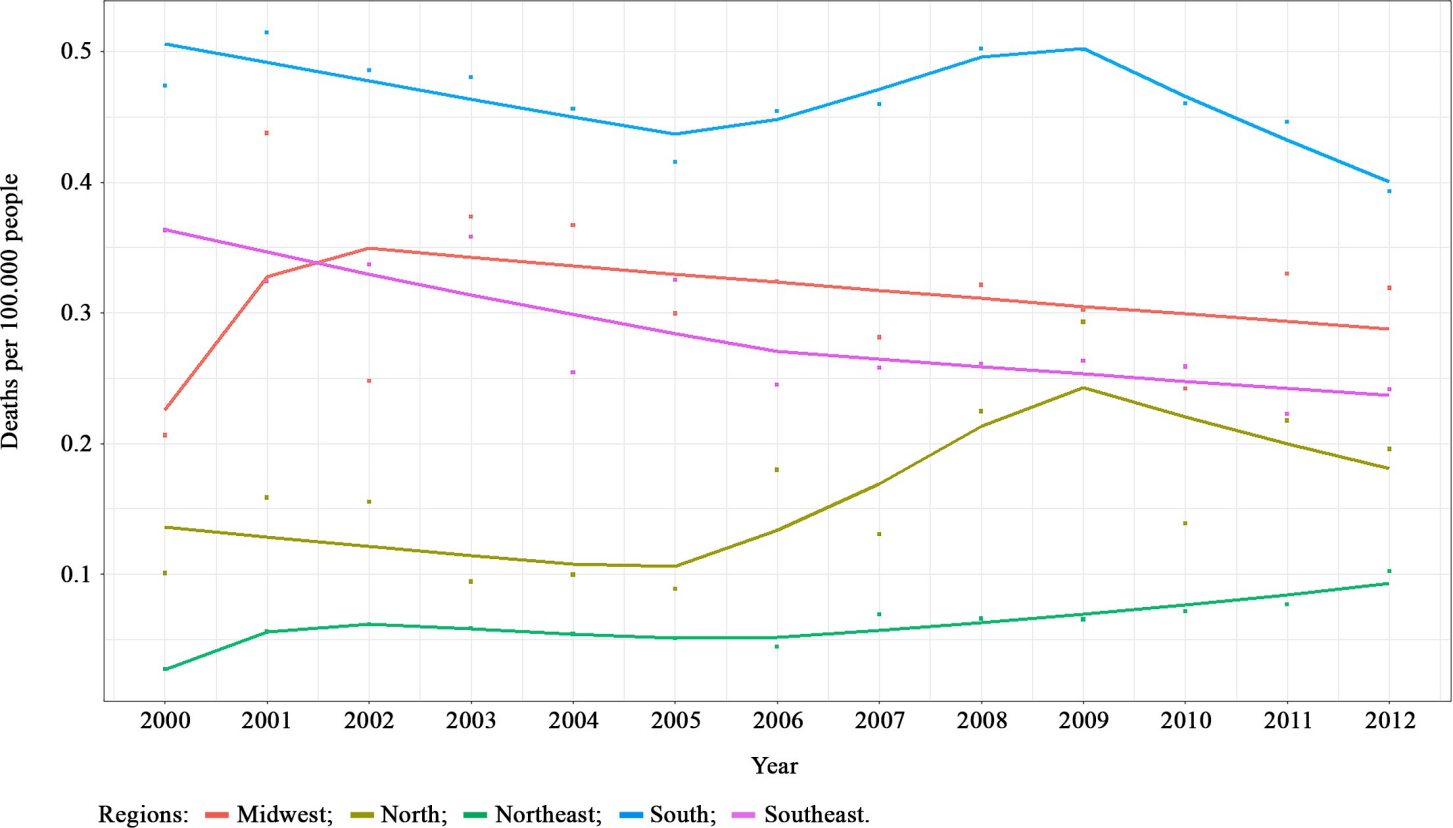
Joinpoint analysis of Cryptococcosis mortality rates according to Brazilian regions between 2000 and 2012 (Total cause of death). Note: Squared points are the observed mortality rates, line represents the expected mortality rates predicted by a Poisson segmented regression.

Total mentioned cause of death by cryptococcosis shows that the South Brazilian region has the highest rates, followed by the Midwest and Southeast. The North and Northeast had the lowest rates (Fig 3). The Southeast, Northeast and South showed significant time trends in mortality rates (S2 Table). The Southeast region showed a decreasing trend of mortality rate (-4.82%) in years 2000–2006. The Northeast region showed 105.20% of increasing trend in 2001 and 10.35% of increasing between 2005 and 2012. Meanwhile, the South region showed decreasing trends of mortality rates in 2000–2005 (-2.89%) and 2009–2012 (-7.27%).

**Fig 4:**
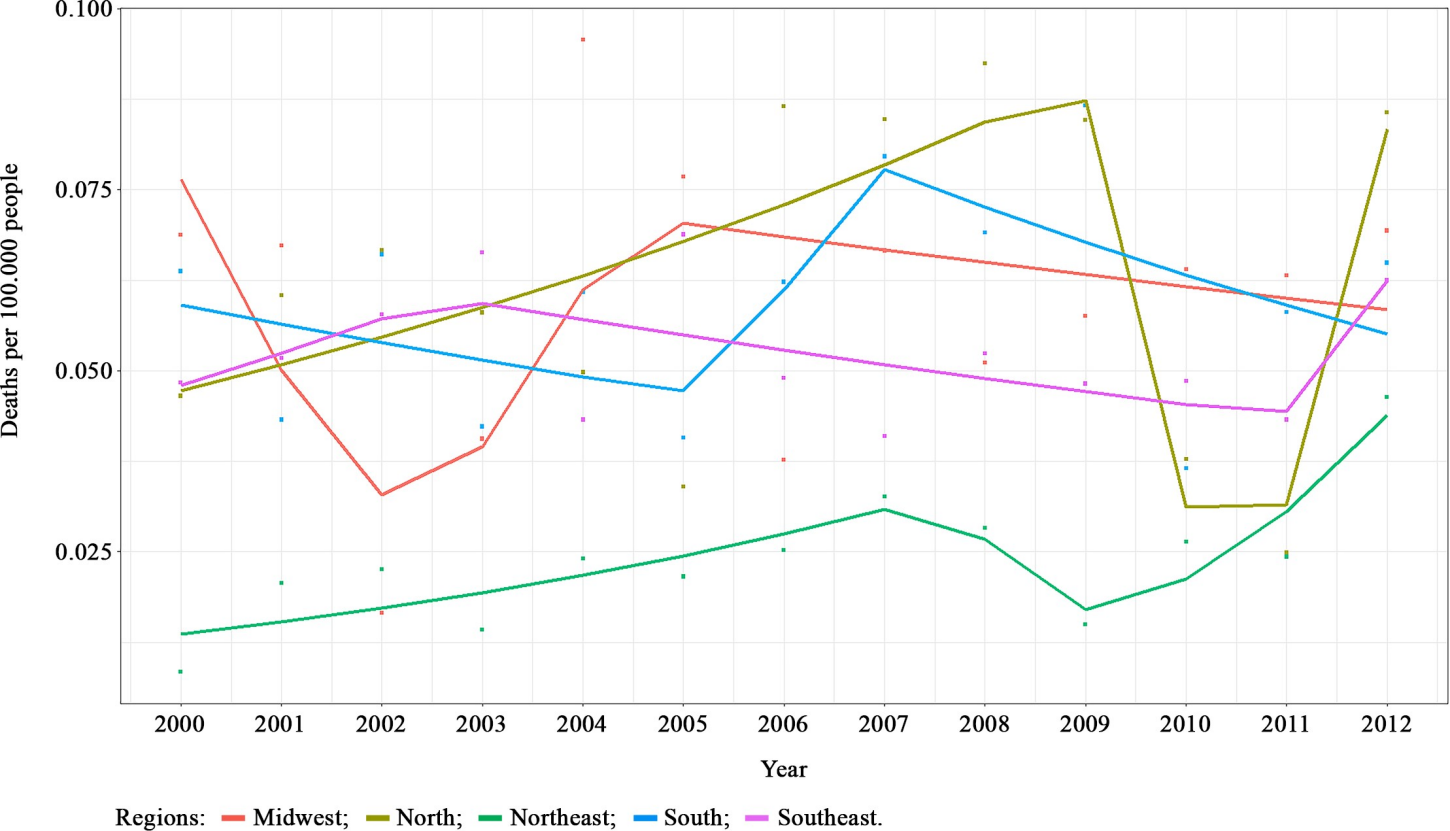
Joinpoint analysis of Cryptococcosis mortality rates according to Brazilian regions between 2000 and 2012 (Basic cause of death). Note: Squared points are the observed mortality rates, line represents the expected mortality rates predicted by a Poisson segmented regression.

The basic cause of death by cryptococcosis show that the Northeast region had the lowest rates (Fig 4). The North and Northeast showed significant time trends in mortality rates (S3 Table). The North region showed a decreasing trend of mortality rate (-64.28%) between years 2009–2010. The Northeast region showed 12.42% of increasing trend between 2000 and 2008 and 43.92% of increasing between 2009 and 2012.

## Discussion

This study points to cryptococcosis as a neglected, severe and often fatal opportunistic condition, since the vast majority of deaths (80%) is hidden by a serious immunosuppressive disease, especially AIDS. In fact, Two patterns of infection were revealed: 1) primary cryptococcosis and 2) opportunistic cryptococcosis, both expressed mainly in the form of meningoencephalitis, indicating late diagnosis, ineffective treatment and difficult access to the national care network.

The study of cryptococcal mortality considering only the basic cause presented limitations, since the presence of underlying immunodeficiencies predominates in the scenario. Thus, when the total causes mentioned in the death certificates was considered, a broad picture of the mycosis in Brazil was revealed, leading to an important reflection on the neglected diseases associated with a host with immunodeficiency. Eighty percent of cryptococcosis deaths were revealed through this approach [25]. In addition, the poorer regions of the north and northeast of Brazil still have high proportions of deaths due to ill-defined causes, which may hide both cryptococcosis and other AIDS-related infectious causes of death [25–27].

The distribution of cryptococcosis deaths according to gender and by all mentioned causes showed a preponderance among males. When associated with AIDS (71.2%), crypto mortality was greater than the mortality due to cryptococcosis as basic cause (62.3%), which corresponds with data from the literature [16, 21].

We observed in primary cryptococcosis an age-matched progressive curve from childhood to adulthood, consistent with progressive environmental exposure to the agent. However, the age-related pattern of cryptococcosis associated with AIDS reflects the predominance of this risk factor, specially in the age group of 20 to 59 (Tables 1 and 2).

In the over 60 age group within the total number of deaths an important differential was also revealed: AIDS-related deaths accounted for 2.7% of the total, while deaths due to cryptococcosis as basic cause represented about 20%, i.e. about seven times higher and, deaths due to cryptococcosis associated with other risk factors represented 33.6%, that is, twelve times higher. This set of evidence seems to corroborate the double profile of cryptococcosis (Table 1).

This age-related profile is consistent with recent reports showing individuals affected by cryptococcal meningitis caused by *C*. *gattii*, with high lethality rates (30 up to 50%) and frequent relapses in the North and Northeast regions of Brazil, along with increased frequency of cryptococcosis in AIDS in young male adults, the involvement of immunocompetent children, adolescents and young adults and the involvement of elderly individuals [15,16,28,29].

The mortality rate of infectious diseases which are difficult to diagnose and that require specialized care, usually expresses the tip of an iceberg. This study hypothesized cryptococcosis as an underestimated *causa mortis*, because the laboratory resources for timely agent identification and with the needed accuracy are often unavailable. The lack of extensive diagnostic laboratory coverage is evident, given that 75% of all meningitis-related deaths had no defined etiology [27].

In this study, the vast majority of cryptococcosis deaths, according to the total number of mentions, was due to cryptococcal meningitis (82.4%), and when associated with AIDS, caused 83.6% of deaths. In Brazil, studies have shown the relevance of cryptococcosis as the main mycosis associated with AIDS death [21], and as the second cause of mortality among systemic mycoses [20]. Cryptococcal meningitis was the second most frequent opportunistic neurological infection in HIV/AIDS [28–30], only surpassed by neurotoxoplasmosis [31–33].

Further, between 1980 and 2002, about 13,000 individuals had cryptococcosis at the time of diagnosis of HIV infection, six percent of the 215,810 registered cases of AIDS in Brazil [22]. Brazilian autopsy studies of the Central Nervous System of AIDS patients showed high cryptococcal involvement: 12% (17/138), 13.5% (34/252) and 15.8% (45/284), respectively [31–33].

The high lethality of cryptococcal meningitis in Brazil results from the convergence of factors such as late suspicion and diagnosis, difficult access to care network, unavailability of rapid laboratory tests, together with inadequate or unavailable antifungals. The screening of Cryptococcal Antigen (CrAg) in HIV infected persons with CD4 count below 100 cells/mm^3^ is highly recommended by the WHO [34–36]. According to international advised protocol to reduce mortality by cryptococcosis, another important issue regarding treatment is to associate 5-flucytosine with amphotericin as a combined initial therapy [37,38], but is as yet unavailable in the national therapeutic arsenal, despite institutional efforts to import this drug [39].

It is worth noting that the mortality rate related to cryptococcal meningitis was higher than that of *Toxoplama* CNS infection (neurotoxoplasmosis), as well as higher than meningitis caused by all viral infections and by tuberculosis. In Africa, cryptococcal meningitis is the most common cause of meningitis in adults [6]. In the US, cryptococcal meningitis hospitalizations were more frequent than all bacterial meningitides combined, with an incidence of 1.1 per 100,000 inhabitants versus 0.728 per 100,000 respectively [38].

This study detected two patterns for cryptococcosis in Brazil: the first, a primary cryptococcosis drawn by deaths recorded as the basic cause, an emerging disease and the second, a cryptococcosis registered as an associated cause of death, an opportunistic infection affecting individuals who present some immunodepression, mainly AIDS-related patients [8,40].

*C*. *gattii* species complex occurs in tropical, subtropical and temperate areas, affecting mainly apparently healthy hosts in contact with environmental sources of infection [8]. *C*. *neoformans* complex is cosmopolitan and affects mainly individuals who present some immunodepression [8,40]. The geographic distribution of *C*. *gattii* in Brazil shows a higher prevalence in the North and Northeast regions compared with the other regions [18,19], while *C*. *neoformans* is more prevalent in the South and Southeast regions [14].

In our study, we did not find significant differences between cryptococcal deaths in the North and Northeast regions, but we found a great difference in the South, Southeast and Central-West regions, where crypto deaths as associated cause were more frequent than basic cause. Furthermore, the majority of individuals infected by HIV in Brazil were concentrated in the South and Southeast regions [41], reinforcing the two profiles of cryptococcosis: the South, Southeast and Central-West with predominant opportunistic infection by *C*. *neoformans* and the North and Northeast with, side by side, the opportunistic infection by *C*. *neoformans* and the primary infection by *C*. *gattii* [14,18,19].

The geographic distribution and joinpoint analysis show that the highest mortality rates due to cryptococcosis reported as basic cause was observed in the North, folowed by the Central West and the South. The state of Mato Grosso, Pará, Mato Grosso do Sul, Amazonas and Santa Catarina showed the highest rates by state. These regions are economically heavily based on agricultural activity. The North and Central West are the new Brazilian agricultural frontiers with intensive population mobility [42,43]. As previously pointed out, recent studies documented the presence of an endemic primary cryptococcosis in the Amazon region, the north, and northeast of Brazil [15–17,19].

The geographic distribution of mortality rates due to cryptococcosis as associated cause evidenced that the highest mortality rates occurred in the most economically dynamic regions of the country. These rates occurred in the South, Central West and Southeast. The highest rates were reported in the states of Santa Catarina, Rio Grande do Sul and Mato Grosso do Sul. This distribution is analogous to the distribution of AIDS deaths in the period, which it is also consistent with the interiorization spreading of the AIDS epidemic in Brazil [41].

The major limitation of this study is in relation to the use of secondary data, which underestimates the true number of deaths related to neglected diseases. The lack of specialized laboratories and medical resources in the poorest regions of the country result in a large number of deaths of indeterminate cause. Furthermore, the SIM does not have access to medical records, only to diagnoses reported on death certificates. Therefore, it is impossible to know how the diagnosis of cryptococcosis was made.

## Conclusions

This study is the first one to apply a holistic approach to cryptococcosis mortality in Brazil. It provides needed visibility to cryptococcosis, revealing two distinct profiles, one primary and the other opportunistic associated mainly with AIDS.

The high frequency of deaths by cryptococcosis meningitis and other severe clinical presentations indicates late diagnosis, unavailability of rapid diagnostic methods, lack of effective antifungal treatments, and difficult access to the care network in the country.

This study can support surveillance and improvement actions aimed at preventing many avoidable deaths by this neglected systemic mycosis.

## Supporting information

S1 Table Distribution of deaths and average mortality rates due to infectious and /or parasitic diseases, according to the basic cause. **Brazil, 2000 to 2012.** Source: DATASUS/MS and IBGE; *Avarage mortality rate/million inhabitants.
(DOCX)

S2 Table Annual Percentage Change (APC) of cryptococcosis mortality rates according to Brazilian regions obtained by a Poisson segmented model in 2000–2012 (Total mentioned cause of death). * Significant trend, APC = Annual Percentage Change, CI = Confidence Interval.
(DOCX)

S3 Table Annual Percentage Change (APC) of cryptococcosis mortality rates according to Brazilian regions obtained by a Poisson segmented model in 2000–2012 (Basic cause of death). * Significant trend, APC = Annual Percentage Change, CI = Confidence Interval.
(DOCX)

S4 Table STROBE checklist. (DOC)
