# Supplementary material for: Mortality by cryptococcosis in Brazil from 2000 to 2012: A descriptive epidemiological study
Source: PLoS Negl Trop Dis. 2019 Jul 29;13(7):e0007569. doi: 10.1371/journal.pntd.0007569 (PMC6687200; doi:10.1371/journal.pntd.0007569)
Supplement: S1 Table — Brazil, 2000 to 2012. Source: DATASUS/MS and IBGE; *Avarage mortality rate/million inhabitants. (DOCX) [file pntd.0007569.s001.docx]

| **S1– Distribution of deaths and average mortality rates due to infectious and /or parasitic diseases, according to the basic cause. Brazil, 2000 to 2012.** | | |
| --- | --- | --- |
| **Chronic and recurrent infectious diseases** | **Deaths** | **Mortality *** |
| AIDS | 148902 | 62.27 |
| Tuberculosis | 63702 | 26.64 |
| Chagas disease | 63578 | 26.59 |
| Schistossomiasis | 6807 | 2.85 |
| Leishmaniosis | 3759 | 1.57 |
| Hanseniasis | 2841 | 1.19 |
| Paracoccidioidomycosis | 1900 | 0.79 |
| Syhilis | 1515 | 0.63 |
| Malaria | 1358 | 0.57 |
| Cisticercosis | 1306 | 0.55 |
| Toxoplasmosis | 1156 | 0.48 |
| Other helmintiasis | 1128 | 0.47 |
| Cryptococcosis | 1121 | 0.47 |
| Other mycosis | 752 | 0.31 |
| Candidiasis | 664 | 0.28 |
| Pneumocystosis | 554 | 0.23 |
| Aspergilosis | 243 | 0.10 |
| Histoplasmosis | 194 | 0.08 |
| Coccidioidomycosis | 24 | 0.01 |
| **Subtotal of chronic and recurrent disease** | **301504** | **126,08** |
| Sequelae of infectious/parasitic disease | 7295 | 3.05 |
| **Acute infectious disease** | | |
| Sepsis | 158618 | 66.33 |
| Intestinal infectious diseases | 68470 | 28.23 |
| Viral hepatites | 31182 | 13.04 |
| Other bacterial diseases | 14218 | 5.95 |
| Meningogoccal infection | 6167 | 2.58 |
| Other viral diseases | 5687 | 2.38 |
| Certain zoonotic bacterial diseases | 4972 | 2.08 |
| Dengue | 3546 | 1.48 |
| Other infectious diseases | 1075 | 0.87 |
| Varicella | 1944 | 0.81 |
| Tetanus | 1825 | 0.76 |
| Viral meningitis | 691 | 0.29 |
| Other sexual transmited diseases | 120 | 0.05 |
| **Subtotal for acute diseases** | **299515** | **125.25** |
| **Total for infectious diseases** | **608314** | **254.39** |
| Source: DATASUS/MS and IBGE  *Avarage mortality rate/million inhabitants | | |
