# Supplementary material for: Mortality by cryptococcosis in Brazil from 2000 to 2012: A descriptive epidemiological study
Source: PLoS Negl Trop Dis. 2019 Jul 29;13(7):e0007569. doi: 10.1371/journal.pntd.0007569 (PMC6687200; doi:10.1371/journal.pntd.0007569)
Supplement: S2 Table — * Significant trend, APC = Annual Percentage Change, CI = Confidence Interval. (DOCX) [file pntd.0007569.s002.docx]

| S2 Table – Annual Percentage Change (APC) of Cryptococcosis mortality rates according to Brazilian regions obtained by a Poisson segmented model in 2000-2012 (Total cause of death) | | | | | | | | | | | | | | | | | | |  |
| --- | --- | --- | --- | --- | --- | --- | --- | --- | --- | --- | --- | --- | --- | --- | --- | --- | --- | --- | --- |
| Region | Trend 1 | | | | Trend 2 | | | | | | | Trend 3 | | | | | | |  |
|  | Years | APC | CI(95%) | | Years | | APC | | CI(95%) | | | Years | | APC | | CI(95%) | | |  |
| North | 2000-2005 | -5,63 | 28,04 | 23,77 | | 2005-2009 | | 26,23 | | -9,17 | 75,44 | | 2009-2012 | | -9,35 | | -30,74 | 18,64 | |
| Southeast | 2000-2006* | -4,82 | -8,64 | -0,83 | | 2006-2012 | | -2,20 | | -7,55 | 3,46 | |  | |  | |  |  | |
| Northeast | 2000-2001 * | 105,20 | 21,22 | 247,20 | | 2001-2005 | | -6,20 | | -17,77 | 7,01 | | 2005-2012* | | 10,35 | | 5,10 | 15,87 | |
| Midwest | 2000-2001 | 45,29 | -24,65 | 180,20 | | 2001-2012 | | -1,92 | | -5,73 | 2,04 | |  | |  | |  |  | |
| South | 2000-2005* | -2,89 | -5,25 | -0,47 | | 2005-2009 | | 5,20 | | -1,98 | 12,92 | | 2009-2012* | | -7,27 | | -11,41 | -2,94 | |
| * Significant trend, APC= Annual Percentage Change , CI=Confidence Interval | | | | | | | | | | | | | | | | | | | |
|  |  |  |  |  | |  | |  | |  |  | |  | |  | |  |  | |
