# Supplementary material for: Mortality by cryptococcosis in Brazil from 2000 to 2012: A descriptive epidemiological study
Source: PLoS Negl Trop Dis. 2019 Jul 29;13(7):e0007569. doi: 10.1371/journal.pntd.0007569 (PMC6687200; doi:10.1371/journal.pntd.0007569)
Supplement: S3 Table — * Significant trend, APC = Annual Percentage Change, CI = Confidence Interval. (DOCX) [file pntd.0007569.s003.docx]

| S3 Table- Annual Percentage Change (APC) of Cryptococcosis mortality rates according to Brazilian regions obtained by a Poisson segmented model in 2000-2012 (Basic cause of death) | | | | | | | | | | | | | |  |
| --- | --- | --- | --- | --- | --- | --- | --- | --- | --- | --- | --- | --- | --- | --- |
| Region | Trend 1 | | | | Trend 2 | | | | Trend 3 | | | | |  |
|  | Years | APC | CI(95%) | | Years | APC | CI(95%) | | | Years | APC | CI(95%) | | |
| North | 2000-2009 | 7,50 | -1,02 | 16,75 | 2009-2010* | -64,28 | -86,03 | -8,68 | | 2010-2012 | 164,50 | -10,01 | 677,40 | |
| Southeast | 2000-2003 | 9,17 | -18,41 | 46,07 | 2003-2011 | -3,77 | -9,53 | 2,37 | | 2011-2012 | 40,76 | -19,64 | 146,60 | |
| Northeast | 2000-2008* | 12,42 | 1,63 | 24,36 | 2008-2009 | -36,11 | -74,30 | 58,82 | | 2009-2012* | 43,92 | 0,16 | 106,80 | |
| Midwest | 2000-2003 | -34,42 | -68,29 | 35,63 | 2003-2004 | 54,81 | -55,81 | 442,30 | | 2004-2012 | -2,62 | -14,14 | 10,45 | |
| South | 2000-2005 | -4,48 | -22,82 | 18,21 | 2005-2007 | 29,44 | -51,55 | 245,80 | | 2007-2012 | -6,67 | -19,10 | 7,67 | |
| * Significant trend, APC= Annual Percentage Change , CI=Confidence Interval | | | | | | | | | | | | | | |
