# Supplementary material for: Mortality by cryptococcosis in Brazil from 2000 to 2012: A descriptive epidemiological study
Source: PLoS Negl Trop Dis. 2019 Jul 29;13(7):e0007569. doi: 10.1371/journal.pntd.0007569 (PMC6687200; doi:10.1371/journal.pntd.0007569)
Supplement: S4 Table — (DOC) [file pntd.0007569.s004.doc]

S4. STROBE Statement—Checklist of items that should be included in reports of ***cross-sectional studies***

|  | Item No | Recommendation |
| --- | --- | --- |
| **Title and abstract** | 1 | (*a*) Indicate the study’s design with a commonly used term in the title or the abstract Title page |
|  |  | (*b*) Provide in the abstract an informative and balanced summary of what was done and what was found Abstract, paragraph 2 and 3 |
| Introduction | | |
| Background/rationale | 2 | Explain the scientific background and rationale for the investigation being reported. Introduction, paragraph 7, 8, 9 |
| Objectives | 3 | State specific objectives, including any prespecified hypotheses Introduction, lines 110-129 |
| Methods | | |
| Study design | 4 | Present key elements of study design early in the paper Methods, line 139 |
| Setting | 5 | Describe the setting, locations, and relevant dates, including periods of recruitment, exposure, follow-up, and data collection Methods, lines 143-156 |
| Participants | 6 | (*a*) Give the eligibility criteria, and the sources and methods of selection of participants Not Applicable |
| Variables | 7 | Clearly define all outcomes, exposures, predictors, potential confounders, and effect modifiers. Give diagnostic criteria, if applicable Methods, lines 157-167 |
| Data sources/ measurement | 8* | For each variable of interest, give sources of data and details of methods of assessment (measurement). Describe comparability of assessment methods if there is more than one group Methods, 143-156, |
| Bias | 9 | Describe any efforts to address potential sources of bias Not Applicable |
| Study size | 10 | Explain how the study size was arrived at Not Applicable |
| Quantitative variables | 11 | Explain how quantitative variables were handled in the analyses. If applicable, describe which groupings were chosen and why Methods, 168-178 |
| Statistical methods | 12 | (*a*) Describe all statistical methods, including those used to control for confounding Methods, lines 168-196 |
|  |  | (*b*) Describe any methods used to examine subgroups and interactions Methods, lines 179-196 |
|  |  | (*c*) Explain how missing data were addressed Not Applicable |
|  |  | (*d*) If applicable, describe analytical methods taking account of sampling strategy Not Applicable |
|  |  | (*e*) Describe any sensitivity analyses Not Applicable |
| Results | | |
| Participants | 13* | (a) Report numbers of individuals at each stage of study—eg numbers potentially eligible, examined for eligibility, confirmed eligible, included in the study, completing follow-up, and analysed Results, lines 198-203 |
|  |  | (b) Give reasons for non-participation at each stage Not Applicable |
|  |  | (c) Consider use of a flow diagram - |
| Descriptive data | 14* | (a) Give characteristics of study participants (eg demographic, clinical, social) and information on exposures and potential confounders Results, Table 1 |
|  |  | (b) Indicate number of participants with missing data for each variable of interest Not Applicable |
| Outcome data | 15* | Report numbers of outcome events or summary measures Results, all tables |
| Main results | 16 | (*a*) Give unadjusted estimates and, if applicable, confounder-adjusted estimates and their precision (eg, 95% confidence interval). Make clear which confounders were adjusted for and why they were included Results, Table 2 |
|  |  | (*b*) Report category boundaries when continuous variables were categorized Results, table 2 |
|  |  | (*c*) If relevant, consider translating estimates of relative risk into absolute risk for a meaningful time period Not Applicable |
| Other analyses | 17 | Report other analyses done—eg analyses of subgroups and interactions, and sensitivity analyses Not Applicable |
| Discussion | | |
| Key results | 18 | Summarise key results with reference to study objectives Discussion, lines 285-290 |
| Limitations | 19 | Discuss limitations of the study, taking into account sources of potential bias or imprecision. Discuss both direction and magnitude of any potential bias Discussion, lines 393-398 |
| Interpretation | 20 | Give a cautious overall interpretation of results considering objectives, limitations, multiplicity of analyses, results from similar studies, and other relevant evidence Conclusions |
| Generalisability | 21 | Discuss the generalisability (external validity) of the study results Not Applicable |
| Other information | | |
| Funding | 22 | Give the source of funding and the role of the funders for the present study and, if applicable, for the original study on which the present article is based Not at manuscript, but in appropriated local of submission program |

*Give information separately for exposed and unexposed groups.

**Note:** An Explanation and Elaboration article discusses each checklist item and gives methodological background and published examples of transparent reporting. The STROBE checklist is best used in conjunction with this article (freely available on the Web sites of PLoS Medicine at http://www.plosmedicine.org/, Annals of Internal Medicine at http://www.annals.org/, and Epidemiology at http://www.epidem.com/). Information on the STROBE Initiative is available at www.strobe-statement.org.
